# Obif, a Transmembrane Protein, Is Required for Bone Mineralization and Spermatogenesis in Mice

**DOI:** 10.1371/journal.pone.0133704

**Published:** 2015-07-24

**Authors:** Koji Mizuhashi, Taro Chaya, Takashi Kanamoto, Yoshihiro Omori, Takahisa Furukawa

**Affiliations:** 1 Laboratory for Molecular and Developmental Biology, Institute for Protein Research, Osaka University, 3–2 Yamadaoka, Suita, Osaka, Japan; 2 Department of Developmental Biology, Osaka Bioscience Institute, 6-2-4 Furuedai, Suita, Osaka, Japan; 3 Department of Orthopedic Surgery, Osaka University Graduate School of Medicine, 2–2 Yamadaoka, Suita, Osaka, Japan; 4 Japan Science and Technology Agency, Core Research for Evolutional Science and Technology, 3–2 Yamadaoka, Suita, Osaka, Japan; Université de Lyon—Université Jean Monnet, FRANCE

## Abstract

**Background:**

Various kinds of transmembrane and secreted proteins play pivotal roles in development through cell-cell communication. We previously reported that *Obif* (Osteoblast induction factor, Tmem119), encoding a single transmembrane protein, is expressed in differentiating osteoblasts, and that *Obif^−/−^* mice exhibit significantly reduced bone volume in the femur. In the current study, we characterized the Obif protein and further investigated the biological phenotypes of a variety of tissues in *Obif^−/−^* mice.

**Results:**

First, we found that O-glycosylation of the Obif protein occurs at serine residue 36 in the Obif extracellular domain. Next, we observed that *Obif^−/−^* mice exhibit bone dysplasia in association with significantly increased osteoid volume per osteoid surface (OV/OS) and osteoid maturation time (Omt), and significantly decreased mineral apposition rate (MAR) and bone formation rate per bone surface (BFR/BS). In addition, we observed that *Obif^−/−^* mice show a significant decrease in testis weight as well as in sperm number. By histological analysis, we found that *Obif* is expressed in spermatocytes and spermatids in the developing testis and that spermatogenesis is halted at the round spermatid stage in the *Obif^−/−^* testis that lacks sperm. However, the number of litters fathered by male mice was slightly reduced in *Obif^−/−^* mice compared with wild-type mice, although this was not statistically significant.

**Conclusions:**

Our results, taken together with previous observations, indicate that Obif is a type Ia transmembrane protein whose N-terminal region is O-glycosylated. In addition, we found that *Obif* is required for normal bone mineralization and late testicular differentiation *in vivo*. These findings suggest that *Obif* plays essential roles in the development of multiple tissues.

## Introduction

Vertebrates are characterized by possession of bones and cartilage as major components of the skeletal system. Bone formation occurs through two distinct processes: endochondral ossification, in which a cartilage model is replaced by bone, and intramembranous ossification, in which bones are shaped directly from condensations of mesenchymal cells without a cartilage intermediate [1]. Normal bone remodeling is mediated by a balance of osteoblast and osteoclast activity, whereby bone tissues maintain bone mass and mineral homeostasis [2]. Recent advances have shown the importance of cell-cell communications for osteoblast differentiation, including Notch-Delta, Ephrin-Eph, and Semaphorin-Plexin pathways [2–5]. However, the exact molecular mechanisms of osteoblast differentiation mediated by cell-cell communication have not been well elucidated. In addition, we propose that unidentified membrane proteins essential for cell-cell communication in osteoblast development are still likely to exist.

We previously identified a gene encoding a single transmembrane protein, Obif, which is predominantly expressed in osteoblasts during mouse bone development [6]. The Obif protein contains several O-glycosylation consensus sites between the signal sequence and the transmembrane domain. We observed several strong smeary bands of higher molecular weight than the unmodified Obif molecular weight by Western blot analysis with an anti-Obif antibody using primary cultured calvarial cells containing osteoblast cells and preosteoblastic MC3T3-E1 cell lysates, suggesting that the Obif protein is glycosylated in osteoblasts. We showed that osteoblast differentiation is stimulated when *Obif* is overexpressed and inhibited when *Obif* is knocked down in MC3T3-E1 cells. We then generated *Obif* mutant mice by targeted gene disruption. Micro computed tomography (μCT) analysis revealed that the femur of *Obif* ^−/−^ mice show a significant decrease of cortical thickness as well as cancellous bone volume (BV/TV) at postnatal day 14 (P14) and progressive bone hypoplasia was observed at 8 weeks of age (8 wks) [7]. The expression levels of osteoblast marker genes were significantly reduced in the calvaria of *Obif* ^−/−^ mice at P4. These data suggested that *Obif* plays an important role in bone formation through the regulation of osteoblast development.

Recent findings showed that some molecules playing critical roles in bone development are also involved in spermatogenesis. Osteocalcin is an osteoblast- secreted molecule, and *osteocalcin*-deficient mice showed increased bone formation [8]. A recent study showed that osteocalcin also promotes Leydig cell maturation, and that *osteocalcin*-deficient mice exhibit a decrease in testis and epididymis weights as well as sperm number. These phenotypes are caused by low circulating testosterone levels in *osteocalcin*-deficient mice, suggesting that *osteocalcin* is required for male fertility in mice by promoting testosterone production in Leydig cells. [9]. *GPRC6A* is a candidate as an osteocalcin receptor, which is expressed in osteoblasts and Leydig cells [10, 11]. *GPRC6A* ^−/−^ mice showed osteopenia and reproduction phenotypes similar to those seen in *Osteocalcin* ^−/−^ mice [10]. During spermatogenesis in mammalian testis, spermatogonia differentiate into spermatocytes, which then proceed to round spermatids. Round spermatids undergo an elongation phase, transforming into mature spermatozoa (the sperm cells) [12]. In these spermatogenesis processes, cell-cell communications play important roles [13, 14]. Impairment in any of these spermatogenesis steps can lead to oligozoospermia or azoospermia, which are major causes for male infertility in humans [15].

In the current study, we first biochemically characterized the Obif protein and found that O-glycosylation of Obif protein occurs at serine residue 36 in the Obif extracellular domain. We examined the development of various bones in *Obif* ^−/−^ mice in more detail than in our previous study. We found that bone formation and bone mineralization are reduced in *Obif* ^−/−^ mice. We then investigated biological phenotypes of *Obif* ^−/−^ mice in other tissues, because *Obif* transcripts are significantly detected in the brain, heart, lung, spleen, skeletal muscle, ovary, testis, and epididymis at the adult stage. Interestingly, we found that testis weight and sperm number significantly decreased in *Obif* ^−/−^ mice. We observed *Obif* expression in spermatocytes and spermatids in the developing testis. We found that late testicular differentiation is disturbed in *Obif* ^**−/−**^ mice, revealing the possibility that *Obif* plays a role in the development of normal male fertility.

## Results

### Obif protein is O-glycosylated at serine residue 36

**Fig 1:**
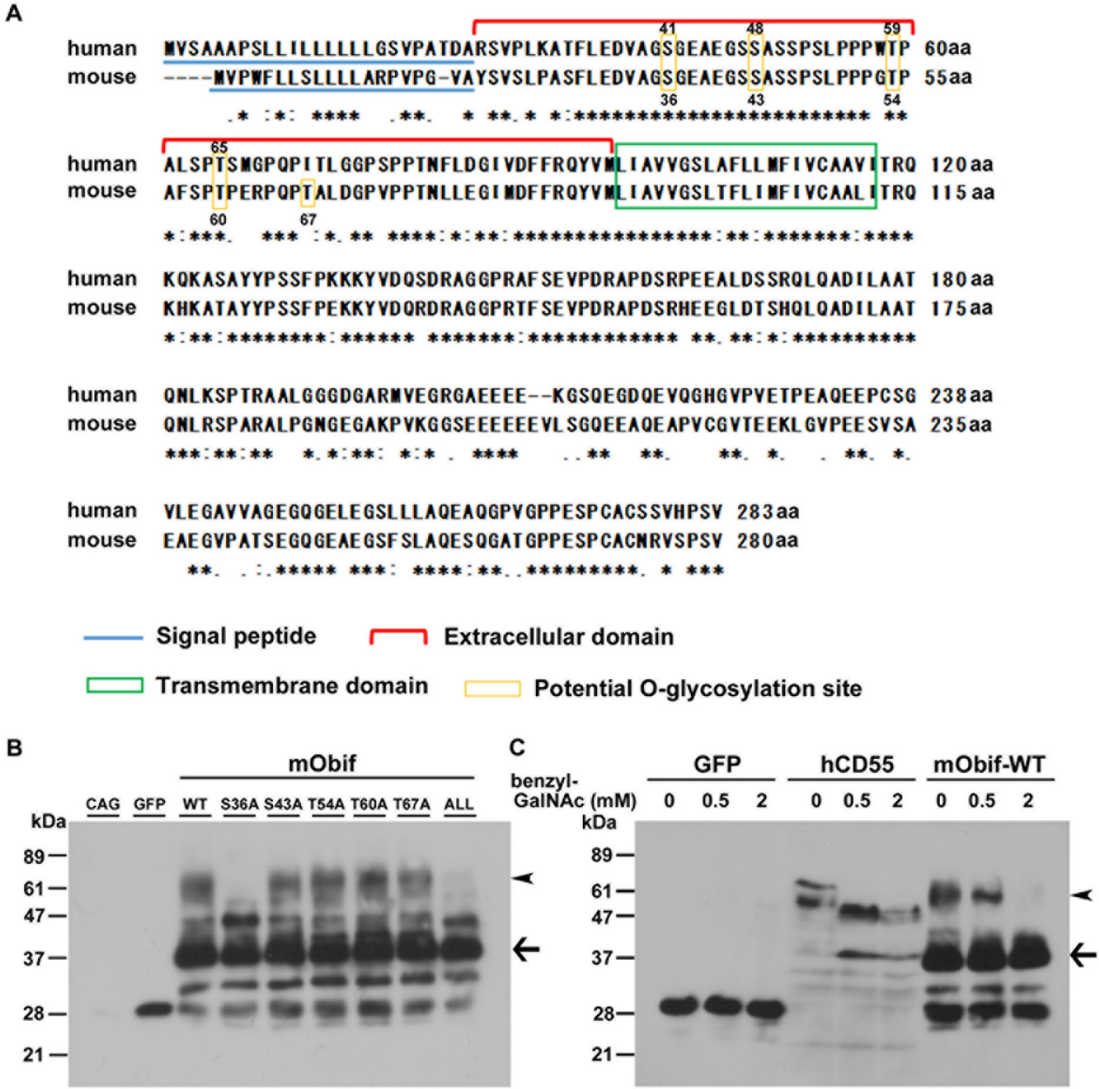
Obif protein is O-glycosylated at serine residue 36. **(A)** Potential O-glycosylation sites of human and mouse Obif proteins. The predicted amino acid sequences of human OBIF (NP_859075.2) and mouse Obif (NP_666274.1) were aligned by the ClustalW program (http://clustalw.ddbj.nig.ac.jp/). Asterisks indicate identical amino acids. Colons and periods indicate similar amino acids. Yellow boxes indicate potential O-glycosylation sites. Red bracket indicates extracellular domain. Blue underline indicates N-terminal signal peptides. Green box indicates single transmembrane domain. (**B-C**) Analysis of O-glycosylation sites in the mouse Obif protein. Constructs of pCAGGS expression vector (CAG), FLAG-tagged GFP (GFP), or FLAG-tagged mObif with or without mutation(s) (wild-type (WT), S36A, S43A, T54A, T60A, T67A, or S36A/S43A/T54A/T60A/T67A (ALL)) were transfected into HEK293T cells. The HEK293T cells were cultured for 24 h. The cell lysates were analyzed by Western blotting using an anti-FLAG M2 antibody **(B)**. FLAG-tagged constructs expressing GFP (GFP), human CD55 (hCD55), or wild-type mouse Obif (mObif-WT) were transfected into HEK293T cells cultured in standard medium. The HEK293T cells were cultured for 24 h, and subsequently cultured for 3 days in medium with or without benzyl-GalNAc. The cell lysates were analyzed by Western blot analysis using the anti-FLAG M2 antibody **(C)**. Arrowheads indicate the 60 kDa band of O-glycosylated mObif. Arrows indicate the 37 kDa band is a nascent form of mObif-WT. Benzyl-GalNAc, benzyl 2-acetamido-2-deoxy- α-D-galactopyranoside, an O-glycosylation inhibitor.

In our previous study, we identified five potential O-glycosylation sites (serine residues 36 (S36) and S43; and threonine residues 54 (T54), T60, and T67) in the mouse Obif protein, using online prediction server NetOglyc 3.1 [16] (Fig 1A), although we did not detect any N-glycosylation consensus site. S36, S43, T54, and T60 are conserved between mouse and human Obif proteins, while T67 is specific to the mouse Obif protein (Fig 1A, yellow boxes). To determine whether the Obif protein is O-glycosylated, we generated constructs expressing a FLAG-tagged full length wild-type mouse Obif (mObif-WT) and FLAG-tagged mutants in which one or all of the five predicted O-glycosylation residues were replaced with alanine residues (mObif-S36A,-S43A,-T54A,-T60A,-T67A, and-ALL). We transfected the constructs into HEK293T cells and the transfected cell lysates were analyzed by Western blot using an anti-FLAG M2 antibody (Fig 1B). Our previous report showed that a band of approximately 37 kDa represented the nascent form of mObif by Western blot analysis with an anti-Obif antibody using primary cultured calvarial cells containing osteoblast cells and preosteoblastic MC3T3-E1 cell lysates [6]. Moreover, we detected several strong smeary bands of higher molecular weight than the unmodified Obif molecular weight in that analysis [6]. We found that the approximately 60 kDa bands in the S36A- or ALL-transfected cell lysates were markedly fainter than those of mObif-WT-transfected cell lysates (Fig 1B, arrowheads), while the band pattern was unaltered in the other mutant-transfected cell lysates (S43A, T54A, T60A, T67A) compared with mObif-WT-transfected cell lysates. Then, to confirm that the Obif protein modification is O-glycosylation, we transfected mObif-WT into HEK293T cells with or without a specific O-glycosylation inhibitor, benzyl 2-acetamido-2-deoxy-α-D-galactopyranoside (benzyl-GalNAc), and analyzed them by Western blot (Fig 1C). We produced constructs expressing FLAG-tagged GFP (GFP) as a negative control and FLAG-tagged human CD55 (hCD55), which encodes a glycosylphosphatidylinositol (GPI)-anchored membrane protein, as a positive control for benzyl-GalNAc treatment [17]. In GFP-transfected cell lysates, we detected a single band of approximately 30 kDa in the presence of 2 mM benzyl-GalNAc. A previous report showed that a band of approximately 70 kDa represented the mature form of hCD55, and a band of approximately 40 kDa represented the nascent form of hCD55 [18]. In the hCD55- transfected lysates, we detected an approximately 70 kDa band of hCD55 in the absence of benzyl-GalNAc. In contrast, in the presence of benzyl-GalNAc, the ~70 kDa band in the hCD55-transfected lysate was undetectable and the 40 kDa band markedly increased, indicating that hCD55 is an O-glycosylated protein as previously reported. In mObif-WT-transfected lysates, we detected an approximately 60 kDa band, the expected mObif-WT size, in the absence of benzyl-GalNAc. On the other hand, we found that the 60 kDa band in the presence of 0.5 mM benzyl-GalNAc became weaker than that in the absence of benzyl-GalNAc (Fig 1C, arrowheads). The 60 kDa band of mObif-WT was undetectable in the cell lysates treated with 2 mM of benzyl-GalNAc. Taken together, these results suggest that Obif protein is O-glycosylated at serine 36.

### O-glycosylation of mObif protein had no effect on mineralization of MC3T3-E1 cells

To investigated whether O-glycosylation of Obif protein affects osteoblast mineralization using MC3T3-E1 cell lines that have the capacity to form mineralized nodules upon stimulation with ascorbic acid and β-glycerophosphate [19], we generated retrovirus constructs expressing a FLAG-tagged full-length wild-type mouse Obif (mObif-WT-Nolan GFP) and FLAG-tagged S36 mutants including mObif-S36A- Nolan GFP and mObif-ALL Nolan GFP (mObif-S36A/S43A/T54A/ T60A/T67A Nolan GFP). Using retroviruses derived from these constructs, we established MC3T3-E1 cells expressing mObif-WT or S36 mutant proteins under mineralizing conditions. Mineralized nodules were assessed after 21 days in the culture by staining with Alizarin red. The Alizarin red staining was eluted and the acid soluble calcium bound dye was quantified (S1 Fig). We examined the effect of mObif S36 mutant proteins on the induction of matrix mineralization, and found that the degree of mineralization was unaltered between wild-type and S36 mutant MC3T3-E1 cells.

### Loss of *Obif* impaired bone growth

We previously examined the *in vivo* role of *Obif* in bone development by bone microstructure imaging using high-resolution μCT on the distal femur of *Obif* ^**−/−**^ mice, and found that cortical thickness as well as cancellous BV/TV significantly decreased in the *Obif* ^−/−^ femur compared with those in wild-type femur [7]. These results showed that *Obif* is required for normal femur and calvaria formation, however, whether the formation of bones other than the femur and calvaria was affected and how *Obif* is required for bone formation were unexplored.

**Fig 2:**
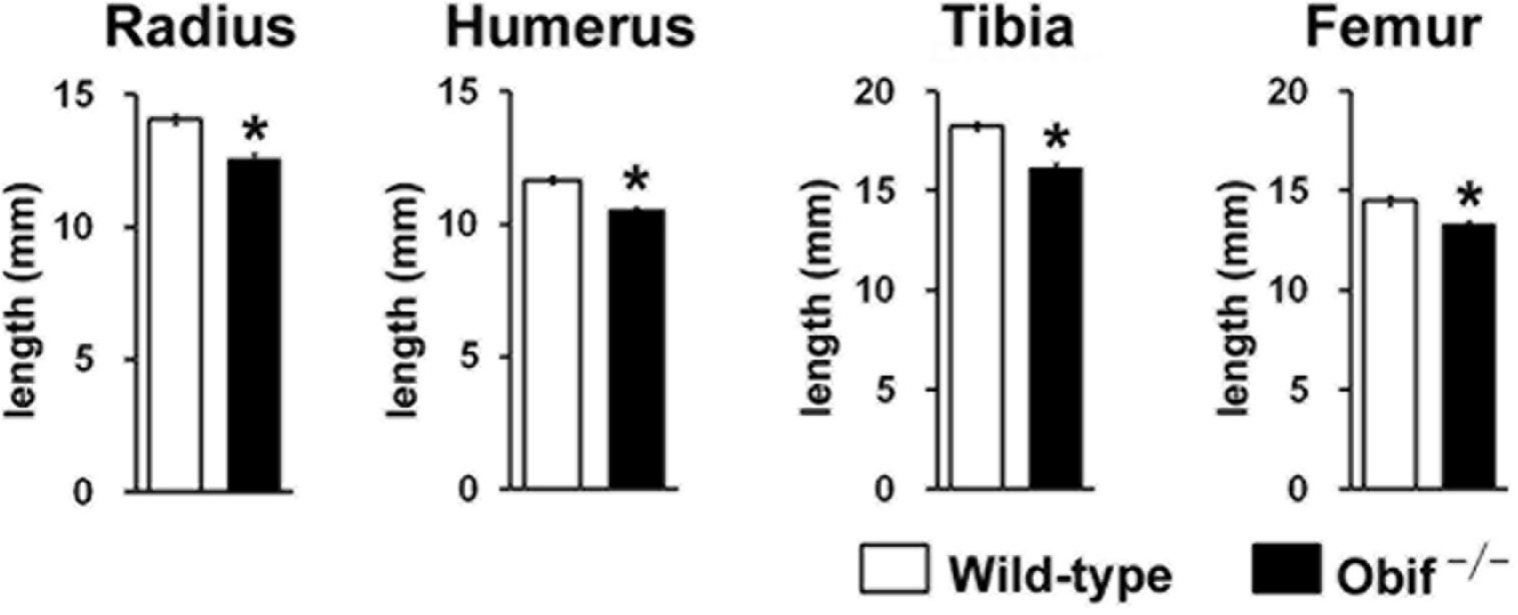
Loss of *Obif* impaired bone growth. **(A)** CR lengths from wild-type (white box) and *Obif* ^−/−^ mice (black box) at 8 wks. **(B)** Longitudinal bone lengths of radius, humerus, tibia, and femur in wild-type (white box) and *Obif* ^−/−^ mice (black box) at 8 wks. CR length, crown-rump length. Error bars show the SEM (n = 5). *P < 0.05.

To investigate bone development in *Obif* ^**−/−**^ mice more extensively, we measured longitudinal bone lengths of the radius, humerus, tibia, and femur at 8 wks (Fig 2). We compared the bone length of the radius, humerus, tibia, and femur, which represent endochondral ossification, between wild-type and *Obif* ^**−/−**^ mice (Fig 2). All of the bone lengths of the radius, humerus, tibia, and femur in *Obif* ^−/−^ mice were significantly shorter than those in wild-type mice. These data suggest that bone development is widely impaired in *Obif* ^−/−^ mice.

### *Obif* ^−/−^ mice showed abnormal bone formation and bone mineralization by bone histomorphometry analysis

**Fig 3:**
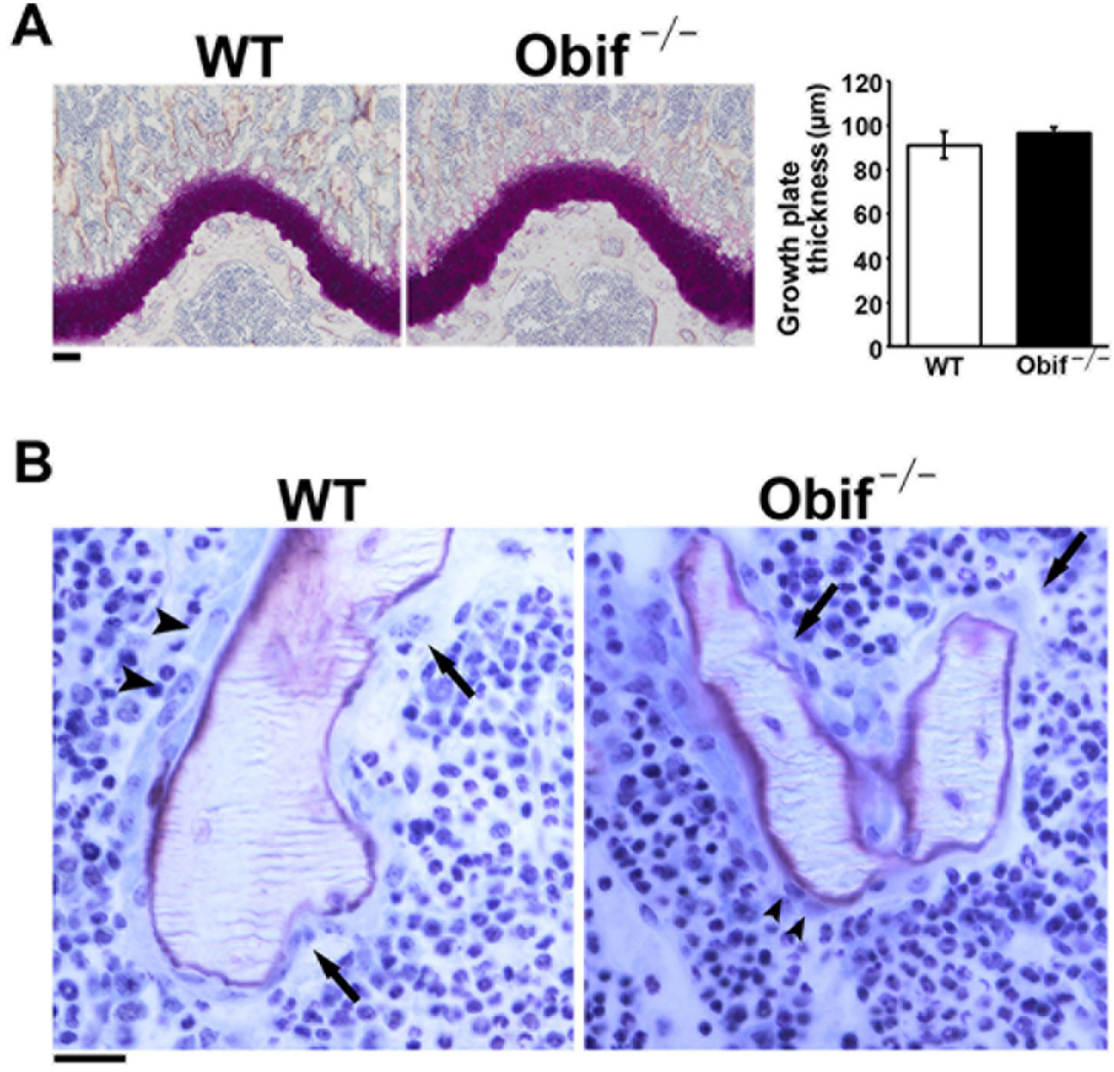
Histological analysis of distal femoral epiphysis of *Obif* ^−/−^ mice. **(A-B)** Villanueva bone staining of distal femur sections from wild-type and *Obif* ^−/−^ mice. The thickness of distal femoral growth plates was unaltered between wild-type (white box) and *Obif* ^−/−^ mice (black box) **(A)**. In wild-type and *Obif* ^−/−^ mice, the osteoblasts (indicated by arrowheads) and osteoclasts (indicated by arrows) were unchanged in number and size. Scale bars represent 100 μm **(A)** and 20 μm **(B)**. Error bars show the SEM (n = 3).

**Fig 4:**
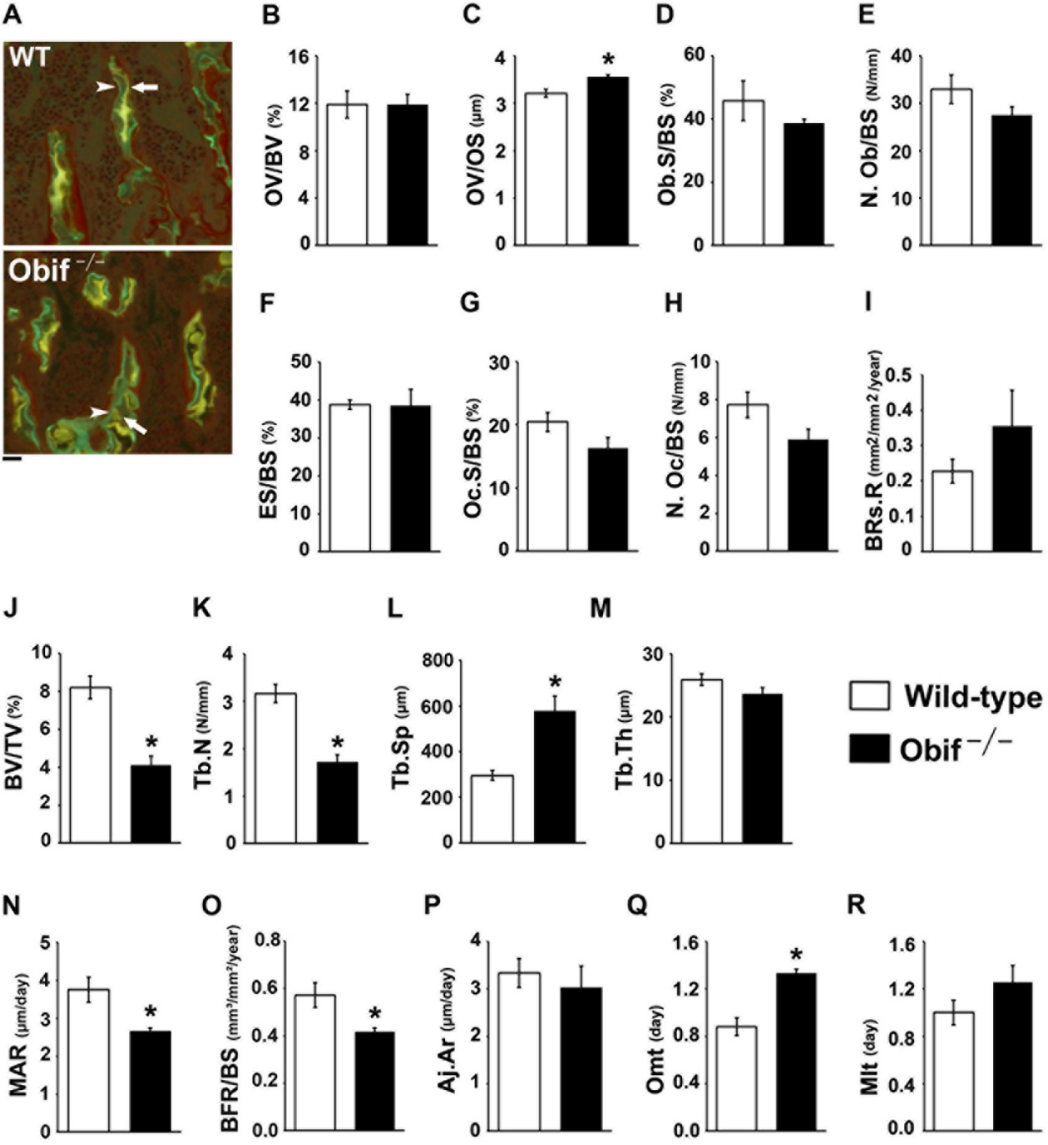
*Obif* ^−/−^ mice showed abnormal bone formation and bone mineralization. **(A)** Fluorescence microscopic images of calcein (indicated by arrowhead) and tetracycline (indicated by arrow) staining of distal femur sections from wild-type and *Obif* ^−/−^ mice. Scale bar represents 100 μm. **(B-R)** Bone histomorphometric analyses of the distal femur sections from wild-type (white box) and *Obif* ^−/−^ mice (black box) at 8 wks. Parameters for bone formation **(B-E)**, bone resorption **(F-I)**, bone volume **(J-M)**, and mineralization **(N-R)** were analyzed. OV/BV, osteoid volume per bone volume; OV/OS, osteoid volume per osteoid surface; Ob.S/BS, osteoblast surface per bone surface; N.Ob/BS, osteoblast number per bone surface; ES/BS, eroded surface per bone surface; Oc.S/BS, osteoclast surface per bone surface; N.Oc/BS, osteoclast number per bone surface; BRs.R, bone resorption rate; BV/TV, bone volume per tissue volume; Tb.N, trabecular number; Tb.Sp, trabecular separation; Tb.Th, trabecular thickness; MAR, mineral apposition rate; BFR/BS, bone formation rate per bone surface; Aj.Ar, adjusted MAR; Omt, osteoid maturation time; Mlt, mineralization lag time. Error bars show the SEM (n = 5). *P < 0.05.

To further investigate the cellular mechanism for how *Obif* affects bone formation, we carried out bone histomorphometry on male femurs isolated from wild-type and *Obif* ^−/−^ mice at 8 wks (Figs 3A, 3B, and 4A–4R). First, we examined wild-type and *Obif* ^−/−^ epiphyseal cartilage and bone by Villanueva bone staining (Fig 3A and 3B). We detected no significant difference in the thickness of distal femoral growth plates between wild-type and *Obif* ^−/−^ mice (Fig 3A). In addition, osteoblasts and osteoclasts were almost unchanged in number and size between wild-type and *Obif* ^−/−^ mice (Fig 3B, arrowheads for osteoblasts and arrows for osteoclasts). Next, to determine several bone parameters in *Obif* ^−/−^ mice, we performed fluorescence microscopic imaging of isolated bone tissues following the injection of calcein and tetracycline into mice (Fig 4A–4R). Fluorescence imaging of calcein and tetracycline, which bind to calcium incorporated into the new bone, enables us to assess various bone parameters, including bone formation, bone resorption, bone mineralization, and bone volume. The bone formation parameters such as osteoid volume/bone volume (OV/BV) showed no significant difference between wild-type and *Obif* ^−/−^ mice, while osteoblast surface/bone surface (Ob.S/BS) and osteoblast number/bone surface (N.Ob/BS) were slightly reduced in *Obif* ^−/−^ mice compared with wild-type mice, but these were not statistically significant (Fig 4B, 4D, and 4E). Moreover, osteoid volume/osteoid surface (OV/OS) significantly increased in *Obif* ^−/−^ mice (Fig 4C). Bone resorption parameters such as eroded surface/bone surface (ES/BS) and bone resorption rate (BRs.R) showed no significant differences between wild-type and *Obif* ^−/−^ mice. While osteoclast surface/bone surface (Oc.S/BS) and osteoclast number/bone surface (N.Oc/BS) were slightly reduced in *Obif* ^−/−^ mice compared with wild-type mice, these were not statistically significant (Fig 4F–4I). In addition, the bone volume parameters such as bone volume per tissue volume (BV/TV) and trabecular number (Tb.N) were decreased and trabecular separation (Tb.Sp) were increased in *Obif* ^−/−^ mice (Fig 4J–4L). Although trabecular thickness (Tb.Th) was slightly reduced in *Obif* ^−/−^ mice compared with wild-type mice, this was not statistically significant (Fig 4M). This observation agrees with our finding obtained by μCT analysis in a previous study [7].

The bone mineralization parameters such as adjusted MAR (Aj.Ar) and mineralization lag time (Mlt) showed no significant differences between wild-type and *Obi* ^f−/−^ mice (Fig 4P and 4R). Interestingly, when we measured mineral apposition rate (MAR), bone formation per bone surface (BFR/BS), and osteoid maturation time (Omt; an indicator of abnormal mineralization), we found that MAR and BFR/BS were significantly decreased and Omt was significantly increased in *Obif* ^−/−^ mice compared with wild-type mice (Fig 4N, 4O and 4Q). These data showed that bone formation and bone mineralization are reduced in *Obif* ^−/−^ bones.

### Bone dysplasia in *Obif* ^−/−^ mice is due to a bone-originating primary cause

It is well known that bone mineralization can be affected by calcium or phosphate abnormalities [20]. To confirm that the bone dysplasia in *Obif* ^−/−^ mice is due to a bone-originating primary cause, we compared serum calcium, phosphate, and 25-OH vitamin D_3_ levels between wild-type and *Obif* ^−/−^ mice (S2A-S2C Fig). Serum calcium, phosphate, and 25-OH vitamin D_3_ levels were unaltered between wild-type and *Obif* ^−/−^ mice (S2A-S2C Fig). In addition, we compared levels of serum osteocalcin, a specific marker of terminal osteoblastic differentiation, between wild-type and *Obif* ^−/−^ mice (S2D Fig). The serum osteocalcin level was significantly decreased in *Obif* ^−/−^ mice compared with wild-type mice. These data suggested that bone dysplasia in *Obif* ^−/−^ mice is due to a bone-originating primary cause rather than indirect bone-originating causes.

### Phenotypic analysis of various *Obif* ^−/−^ mouse tissues

**Fig 5:**
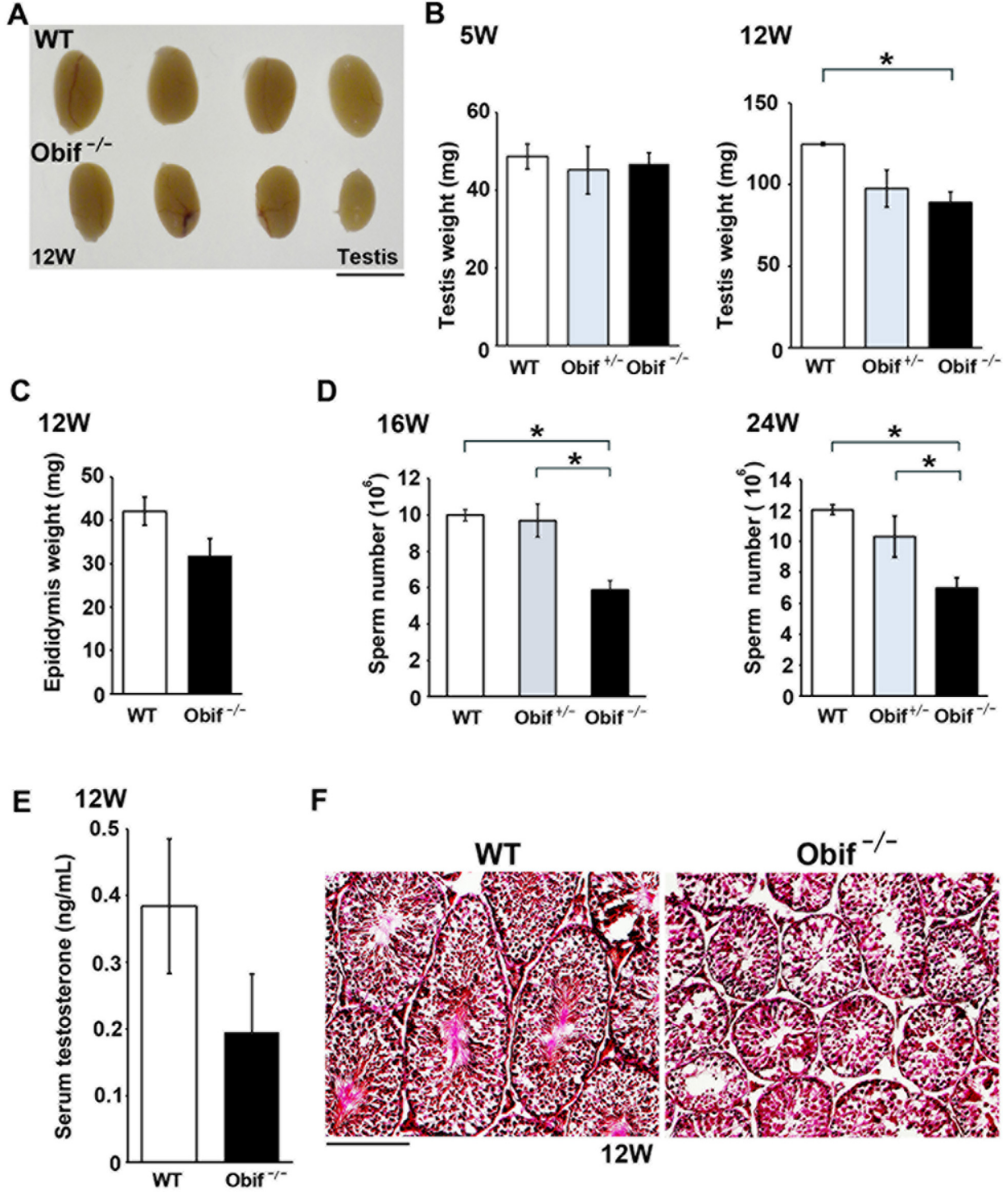
*Obif* is required for normal spermatogenesis. **(A)** Gross appearance of testes in male wild-type and *Obif* ^−/−^ mice at 12 wks. **(B)** Comparison of testicular weight in wild-type (white box), *Obif* ^+/−^ (grey box), and *Obif* ^−/−^ (black box) mice at 5 wks and 12 wks (n = 6). **(C)** Comparison of epididymis weight between wild-type and *Obif* ^−/−^ mice at 12 wks (n = 6). **(D)** Comparison of sperm number from cauda epididymis between wild-type, *Obif* ^+/−^, and *Obif* ^−/−^ at 16 wks and 24 wks (n = 4). **(E)** The level of serum testosterone in wild-type and *Obif* ^−/−^ mice at 12 wks (n = 6). **(F)** H&E staining of testis sections from wild-type and *Obif* ^−/−^ mice at 12 wks. Scale bars represent 5 mm (**A**), and 50 μm **(F)**. Error bars show the SEM. *P < 0.05.

In our previous study, we found that *Obif* transcripts are highly expressed in mouse tissues, including calvaria, brain, and lung by Northern blot analysis [6]. To further examine *Obif* expression in various mouse tissues including the cerebrum, thymus, heart, lung, liver, spleen, kidney, muscle, blood, calvaria, femur, ovary, testis, epididymis, and prostate in wild-type mice at 4 wks, we performed RT-PCR analysis of RNAs isolated from these tissues using *Obif*-specific intron-spanning primers (S3A Fig). Although the *Obif* RNA was undetectable in the blood, the other tissues that we examined produced bands of the expected size for *Obif* (S3A Fig). We then performed phenotypic analysis of *Obif* ^−/−^ tissues, including the brain, heart, lung, spleen, skeletal muscle, ovary, testis, and epididymis (S3B–S3G Fig; Fig 5A–5F). As far as we examined, the gross appearance of the brain, heart, lung, spleen, skeletal muscle, and ovary in *Obif* ^−/−^ mice was normal, compared with those in wild-type mice. To examine the histological integrities of these tissues in *Obif* ^−/−^ mice, we performed toluidine blue and hematoxylin and eosin (H&E) staining on tissue sections prepared from wild-type and *Obif* ^−/−^ mice. For tissue sections isolated from the brain, heart, lung, spleen, skeletal muscle, and ovary in wild-type and *Obif* ^−/−^ mice, we observed no significant abnormality in *Obif* ^−/−^ tissues as far as we examined (S3B-S3G Fig).

### *Obif* is required for normal spermatogenesis

We then examined the testis and epididymis, where *Obif* mRNAs were strongly detected by RT-PCR analysis (S3A Fig). The testes isolated from *Obif* ^−/−^ mice appeared substantially smaller than those from wild-type mice (Fig 5A). When we compared testis weight at 5 wks and 12 wks between wild-type and *Obif* ^−/−^ mice, we found that testis weight at 12 wks was significantly lighter in *Obif* ^−/−^ mice compared with that in wild-type mice, although testis weight at 5 wks was unaltered between wild-type and *Obif* ^−/−^ mice (Fig 5A and 5B). While epididymis weight of *Obif* ^−/−^ mice was slightly lighter compared with that of wild-type mice, it was not statistically significant (Fig 5C). We then counted sperm number in both wild-type and *Obif* ^−/−^ cauda epididymis at 16 wks and 24 wks. We found that sperm number significantly decreased in *Obif* ^−/−^ mice compared with wild-type mice both at 16 wks and 24 wks (Fig 5D). Because testosterone is a well-known inducer of spermatogenesis, we tested whether these phenotypes could be due to a decrease in testosterone level. We compared the level of serum testosterone between wild-type and *Obif* ^−/−^ mice at 12 wks. We found that testosterone levels were statistically unaltered between wild-type and *Obif* ^−/−^ mice, suggesting that oligozoospermia in *Obif* ^−/−^ mice is due not to a secondary cause, but to a primary testicular cause (Fig 5E). In addition, since previous report showed that osteocalcin regulates testosterone biosynthesis in mouse testes [9], we compared serum osteocalcin levels between wild-type and *Obif* ^−/−^ mice. We found that serum osteocalcin levels were significantly reduced in *Obif* ^−/−^ compared with wild-type mice. To investigate histological abnormalities of the testis in *Obif* ^−/−^ mice, we performed H&E staining on testis sections from wild-type and *Obif* ^−/−^ mice at 12 wks. We observed that seminiferous tubules are smaller and appear immature in *Obif* ^−/−^ mice compared with wild-type mice (Fig 5F), suggesting that Obif is essential for normal spermatogenesis.

### *Obif* has a possible role in the development of normal male fertility

To investigate how *Obif* deficiency affects male fertility, we investigated the number of litters, the number of pups per litter, and the number of days until the first litter was born between wild-type male mice mated with wild-type female mice and *Obif* ^−/−^ male mice mated with wild-type female mice (Table 1). Male mice at 12 wks were mated with ICR fertile females at 10 wks for 28 days. There were no significant differences in the number of pups per litter between wild-type and *Obif* ^−/−^ male mice, nor in the number of days until the first litter was born. However, *Obif* ^−/−^ male mice produced a slightly reduced number of litters compared with wild-type mice, although this was not statistically significant (*p* = 0.054, Table 1).

**Table 1:** Fertility parameters.

Genotype	Number of Litters	Number of Pups	Time to First Litter (Days)
WT (n = 10)	1.40 ± 0.16	12.70 ± 0.74	20.50 ± 0.62
*Obif* ^−/−^ (n = 10)	0.90 ± 0.18	10.94 ± 1.33	25.25 ± 2.57

Male mice at 12 wks were placed with ICR fertile females at 10 wks for 28 days. Results are shown as mean ± SEM. All data were analyzed with the *F*-test to determine normality, and the appropriate *t*-test was applied at the level of 5%.

### *Obif* is expressed in spermatocytes and spermatids in the developing testis

Since *Obif* ^−/−^ mice showed impaired spermatogenesis, we then investigated the localization of *Obif* transcripts in the developing testis at P14 by *in situ* hybridization together with testicular differentiation and cellular marker probes in the testis (S4A–S4G Fig). While almost no signal was detected with the *Obif* sense probe, the *Obif* anti-sense probe produced modest but significant staining in the center region of seminiferous tubules (S4A and S4B Fig). To identify *Obif*-expressing cell types, we compared the *Obif*-staining pattern with those of cell type-specific markers. We used cell type-specific marker genes, including *PLZF* (*Promyelocytic leukemia zinc-finger*) for spermatogonia (S4C Fig) [21], *Acrosin* and *Calmegin* for pachytene spermatocytes to round spermatids [22, 23] (S4D and S4E Fig), *3β-HSD* (3β-hydroxysteroid dehydrogenase/Δ5–Δ4 isomerase) for Leydig cells (S4F Fig) [24], and *Sox9* for Sertoli cells (S4G Fig) [25]. We found that the *Obif* expression pattern is similar to that of *Acrosin* or *Calmegin*, suggesting that *Obif* transcripts are expressed in the cells from pachytene spermatocyte to round spermatid stages.

### Testicular differentiation is perturbed in *Obif* ^−/−^ mice

**Fig 6:**
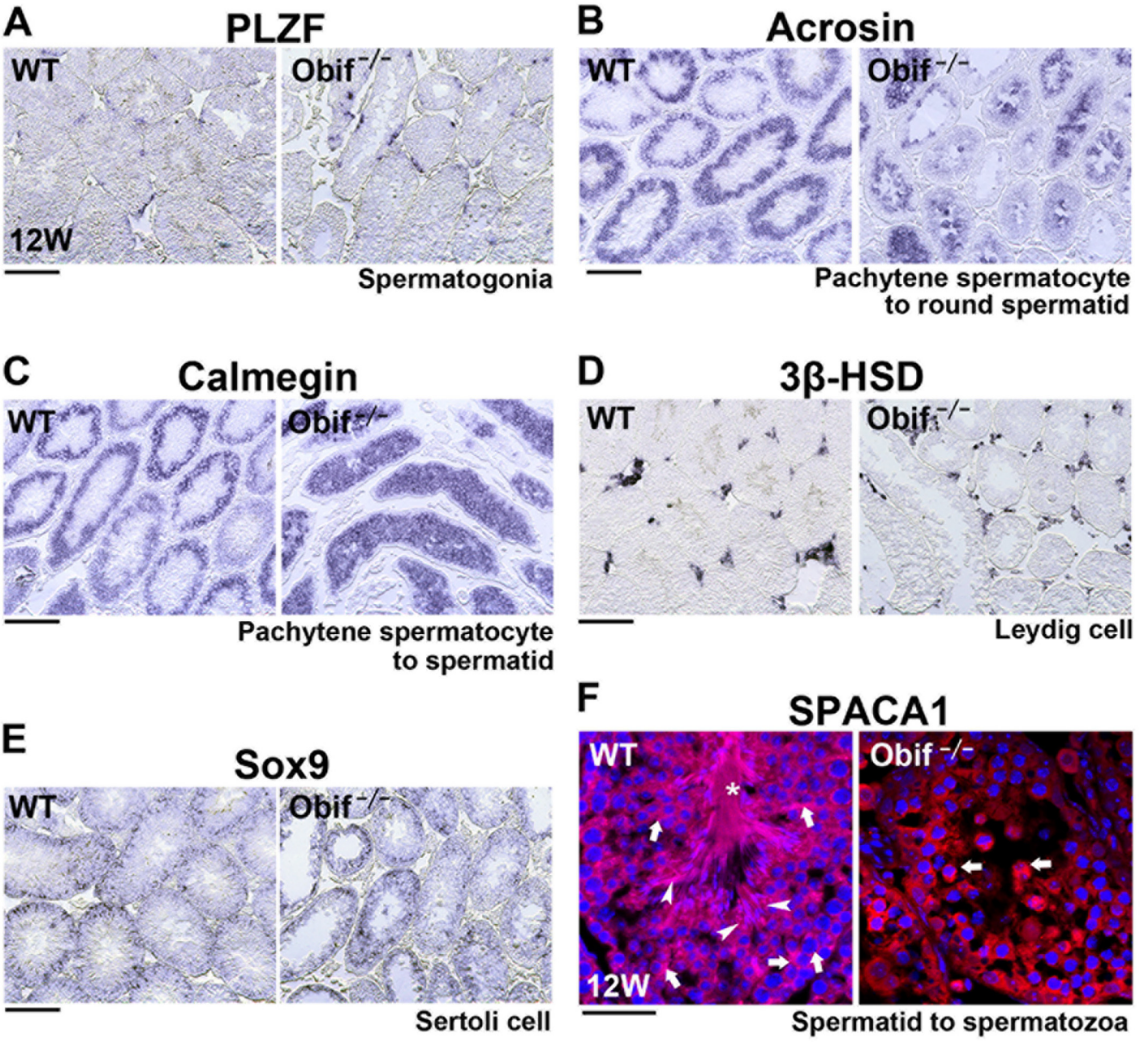
Late spermatogenesis is perturbed in the *Obif* ^−/−^ testis. **(A-E)** *In* situ hybridization analysis for testicular sections from wild-type and *Obif* ^−/−^ mice at 12wks, using probes of *PLZF* **(A)**, *Acrosin* **(B)**, *Calmegin* **(C)**, *3β-HSD* **(D)**, and *Sox9* **(E)**. **(F)** Immunofluorescence staining of testicular sections with an anti-SPACA1 antibody, which detects cells at stages from spermatids to spermatozoa. Arrows, round spermatids; arrowheads, elongated spermatids; asterisks, spermatozoa. Scale bars represent 100 μm **(A-E)** and 20 μm **(F)**.

To investigate how loss of *Obif* affects spermatogenesis, we performed *in situ* hybridization analysis on mature testis sections from wild-type and *Obif* ^−/−^ mice at 12 wks using *PLZF*, *Acrosin*, *Calmegin*, *3β-HSD* and *Sox9* probes (Fig 6A–6E). At this stage, spermatozoa are localized at the center region of seminiferous tubules and spermatogenesis occurs in the peripheral regions of seminiferous tubules. We observed that *PLZF* expression was almost unaltered between wild-type and *Obif* ^−/−^ mice (Fig 6A). Notably, both *Acrosin* and *Calmegin* expression patterns were distinctively affected in the *Obif* ^−/−^ testis compared with that in the wild-type testis. In the *Obif* ^−/−^ testis, *Acrosin* and *Calmegin* were detected in the central region of seminiferous tubules, which are occupied by spermatozoa in the wild-type testis at the adult stage (Fig 6B and 6C). On the other hand, *3β-HSD* expression in Leydig cells and *Sox9* expression in Sertoli cells were unchanged between the wild-type and *Obif* ^−/−^ testis (Fig 6D and 6E). These results suggest that *Obif* regulates testicular parenchymal cell differentiation between pachytene spermatocyte and spermatid stages. In order to further confirm whether the absence of Obif perturbs late spermatogenesis, we performed immunofluorescence staining on testicular sections using an anti-SPACA1 antibody, which detects cells at round spermatid, elongated spermatid, and spermatozoa stages (Fig 6F) [26]. While we observed round spermatids (arrows), elongated spermatids (arrowheads), and spermatozoa (asterisks) in the wild-type testis, we detected only round spermatids, but no elongated spermatids or spermatozoa, in the *Obif* ^−/−^ testis. This result suggests that spermatogenesis is halted at the round spermatid stage in the *Obif* ^−/−^ testis that lack sperm.

## Discussion

We previously identified a gene encoding a single transmembrane protein, Obif, and we showed that *Obif* can promote osteoblastic differentiation with only the N-terminus fragment prior to the membrane domain, suggesting that Obif functions as a ligand in osteoblast differentiation [6]. Furthermore, we generated an *Obif* null allele by targeted gene disruption, and revealed that *Obif* ^−/−^ mice showed a significant decrease of cortical thickness as well as cancellous BV/TV in the femur. In addition, the expression levels of osteoblast marker genes, including *Collagen 1a1*, *Osteopontin*, *Runx2*, and *Osterix*, were significantly reduced in the calvaria of *Obif* ^**−/−**^ mice [7]. These data suggested that *Obif* plays an essential role in bone formation in association with reduced osteoblastogenesis. In the current study, in order to clarify how *Obif* increase volume, we performed bone histomorphometry on *Obif* ^**−/−**^ mice. We found that activities of bone formation and bone mineralization significantly decreased in *Obif* ^**−/−**^ mice, and that the bone mineralization defect in *Obif* ^−/−^ mice is independent of calcium and phosphate metabolisms. These findings suggest that lack of *Obif* impaired bone mineralization through reduced osteoblast activity.

Moreover, we found that *Obif* ^**−/−**^ mice show a decrease in testis weight as well as sperm number. In addition, we found that *Obif* is expressed in spermatocytes and spermatids in the developing testis, and that spermatogenesis is completely halted at the round spermatid stage in the *Obif* ^−/−^ testis that lacks sperm. However, the number of litters fathered by male mice were slightly reduced in *Obif* ^−/−^ mice compared with wild-type mice, although this was not statistically significant. Taken together, these results suggest that *Obif* has a possible role in the development of normal male fertility. Recent studies showed that junctions between Sertoli cells and spermatogenic cells are essential for normal spermatogenesis [27–29]. For example, Junctional adhesion molecule C (Jam-C) on the surface of spermatids binds to Jam-B on the surfaces of Sertoli cells. *Jam-C*-null male mice are infertile due to a failure of round spermatids to differentiate into spermatozoa [27]. Similarly, Nestin-2 protein on the surfaces of Sertoli cells interacts with Nestin-3 protein on the surface of elongated spermatid heads. *Nestin-2*-deficient mice are infertile due to aberrant morphogenesis of spermatids [28]. Considering that Obif is a membrane protein and that Obif expression was detected in spermatocytes and spermatids, we hypothesize that Obif binds to an unidentified partner on Sertoli cells to mediate the cell-cell communication that plays a role in normal testis differentiation.

Interestingly, recent studies have shown that *Obif* transcripts are highly enriched in microglial cells [30–32], however, *Obif* functions in microglial cells remain unknown. Moreover, a large-scale functional screen using mouse knockouts for secreted or transmembrane proteins revealed that *Obif* ^**−/−**^ mice in the *129/B6* mixed genetic background appear to show abnormalities in neurology including the open field test and tail suspension test [33]. However, behavioral analysis using mice with the C57BL/6 genetic background is needed.

In our previous study, we identified several potential O-glycosylation sites in extracellular domain of mouse and human Obif proteins using the online prediction server NetOglyc 3.1 [6]. The surface membranes of mammalian cells are abundant in N- and O-linked glycoproteins which are related to cellular events and closely involved in cell-cell and cell-matrix interactions [34]. To confirm O-glycosylation of the Obif protein, we used an O-glycan inhibitor benzyl-GalNAc, which is widely used in cell culture assays [35–37]. We narrowed down the O-glycosylation site to serine residue 36 in the Obif extracellular domain using benzyl-GalNAc and Obif mutants. This observation suggests that Obif is a type Ia transmembrane protein in which the N-terminal region is O-glycosylated. It should be noted that other studies have reported that *Obif* interacts with Smad-1, -5 and Runx2 transcription factors involved in osteoblastic differentiation [38]. Our observation of Obif glycosylation may not support this mechanism.

Our current and previous studies suggest that the Obif protein functions through cell-cell interactions in bone and testis development. In future studies, it will be important and interesting to identify a partner molecule for Obif in these tissues as well as to elucidate Obif functions in microglial cells.

## Materials and Methods

### Mice

*Obif* ^−/−^ mice in the *129/SvEv* genetic background were generated as previously described [6]. To obtain *Obif* ^−/−^ mice for all experiments *Obif* ^+/−^ male mice were mated with *Obif* ^+/−^ female mice. Experiments were carried out on *Obif*-deficient mice at 5, 8, 12 and 16 wks. ICR strain female mice used for fertility tests were purchased from Japan SLC (Hamamatsu, Japan). In this study, we used 84 mice that were housed in a temperature-controlled room at 22° C with 12 h light/dark cycle. Fresh water and rodent diet were available at all times. All procedures were approved by the Institutional Safety Committee on Recombinant DNA Experiments and Animal Research Committee of Osaka Bioscience Institute, and by the Recombinant DNA (#3380–4) and Animal Experimental Committees (#24-05-1) of Institute for Protein Research, Osaka University, and were performed in compliance with the relevant institutional guidelines. Mice were sacrificed by CO_2_-induced euthanasia, which was performed by placing mice in a container and exposing them to CO_2_. The mice ceased breathing within 30 sec.

### Plasmids constructs

A plasmid encoding full-length mouse Obif was amplified using a FANTOM II clone cDNA [39] (GenBank accession No. BC025600) as a template and inserted into the C-terminal FLAG-tagged pCAGGSII expression vector to produce pCAG-full-length-mouse-Obif-FLAG [6]. The mouse Obif mutations were introduced by PCR using primers containing mutations. The human pME18Sf+-Cd55-FLAG expression vector was kindly provided by Dr. Y. Maeda (Research Institute for Microbial Diseases and WPI Immunology Frontier Research Center, Osaka University). The retroviral vector expressing mouse Obif and Obif mutants were constructed in a pBMN-I-GFP vector that was kindly provided by Dr. Gary Nolan (Stanford University). The mouse Obif or Obif mutations cDNA that were added to the FLAG tag at the C-terminus were inserted into the multicloning site of pBMN-I-GFP (Nolan-GFP).

### Cell culture and transfection with O-glycosylation inhibitor

HEK293T cells [40] were grown in DMEM (Sigma, St. Louis, MO) with 10% FBS. Transfection of plasmid DNA was performed using Lipofectamine LTX (Invitrogen, Carlsbad, CA) for HEK293T cells according to the manufacturer’s instruction. All cells were cultured in a plate with or without benzyl-GalNAc (Sigma-Aldrich, St. Louis, MO) for 3 days with a slight modification of the previously published protocol [41].

### Western blot analysis

Proteins were separated by SDS-polyacrylamide gel electrophoresis (PAGE) and transferred to a polyvinylidene difluoride (PVDF) membrane (ATTO, Tokyo, Japan). The membrane was incubated with a mouse monoclonal anti-FLAG M2 antibody (1:1000; Sigma-Aldrich, F1804) and incubated with a horseradish peroxidase-conjugated goat anti-mouse IgG (1:10,000; Zymed Laboratories, San Francisco, CA). The bands were developed using Chemi-Lumi One L (Nacalai, Kyoto, Japan).

### Cell Culture

Phoenix cells [42] were grown in DMEM (Sigma-Aldrich, St. Louis, MO, USA) with 10% FBS. MC3T3-E1 cells (preosteoblastic cells) were obtained from the Riken Cell Bank (Ibaraki, Japan) and grown in α-MEM (Sigma-Aldrich) containing 10% FBS. To induce mineralization, the media was supplemented with 50 μg/ml of ascorbic acid (Sigma-Aldrich) and 10 mM of β-glycerophosphate (Sigma-Aldrich) as described previously [6].

### Production of recombinant retrovirus and infection

To produce the retroviral particles, the plasmid DNA was transfected along with a helper plasmid into a subline of the 293T cell line, Phoenix cells. The medium was changed 24 h after transfection and supernatant containing the virus was harvested, concentrated, and titered by analyzing the frequency of GFP-positive cells in a number of MC3T3-E1 cells infected with serially diluted viral supernatant. The efficiency of infection was determined at 48 h by the number of GFP-positive cells. MC3T3-E1 cells were maintained for 2 days in mineralizing media, and then infected for 48 h in the presence of polybrene (8 μg/ml), after which cells continued incubation in mineralizing culture media. Cells were maintained in mineralizing media for 3 weeks.

### Alizarin red staining

After 21 days, cultured cells were fixed with 4% paraformaldehyde in PBS buffer. Alizarin red staining was eluted with 28% ammonia solution and the acid soluble solution was measured at OD 415nm.

### RT-PCR

RT-PCR was performed as described previously [43]. In brief, total RNA (1 μg) was isolated from wild-type mice tissues, including cerebrum, thymus, heart, lung, liver, spleen, kidney, muscle, blood, calvaria, femur, ovary, testis, epididymis, and prostate, using TRIzol RNA extraction reagent (Invitrogen). The total RNA was reverse transcribed into cDNA using SuperSript II reagent (Invitrogen) with random hexamers. The cDNAs were used as templates for PCR reactions with rTaq polymerase (Takara, Kyoto, Japan). The following sets of PCR primers were used: 5’-CAGCT-GCACCCGGTCCTTCACCCAGAG-3’ (*Obif*, forward) and 5’-GGAGGTTGGTGG- GTGGCACGGGGCCGT-3’ (*Obif*, reverse), and 5’-CGTGCGTGACATCAAAG- AGAA-3’ (*β-actin*, forward) and 5’-TGGATGCCACAGGATTCCAT-3’ (*β-actin*, reverse).

### Bone histomorphometric analyses

Mice were subcutaneously injected with tetracycline hydrochloride (20 mg/kg; Sigma-Aldrich, 4 days before dissection), and subcutaneously injected with calcein (20 mg/kg; Dojindo, Japan, 2 days before dissection). Bone histomorphometry was performed on undecalcified sections. Femurs were fixed with 70% ethanol, and embedded in glycol-methacrylate. Sections were stained with Villanueva bone stain to identify cellular components. Histomorphometric parameters were measured at Ito Bone Science Institute (Niigata, Japan).

### Histological analysis

To prepare frozen sections of the brain, heart, lung, spleen, skeletal muscle, ovary, and testis, we perfusion-fixed the tissues with 4% paraformaldehyde in PBS buffer and embedded in OCT compound (Sakura Finetechnical, Tokyo, Japan). Frozen sections (10–20 μm thick) were stained with hematoxylin and eosin or toluidine blue, and subjected to histochemical analysis.

### *In situ* hybridization

*In situ* hybridization was performed as described previously [6]. The following sets of PCR primers were used: 5’-AGGAGGGTTTGTGGGCCAGAGGA-3’ (*3β-HSD*, forward) and 5’-ATTAGGGCGGAGCCCCCATTCCT-3’ (*3β-HSD*, reverse), 5’-ACA- GCCGCAGGTACCACGCCTGT-3’ (*Acrosin*, forward) and 5’-GGGCTCAAACGT- GGAGAAGCGGT-3’ (*Acrosin*, reverse), and 5’-CAGGTGCCTGGCGGCGACAG- AGGGCT-3’ (*Calmegin*, forward) and 5’-AGCCCACTGATCGGCCACCTCCT-3’ (*Calmegin*, reverse). We used *Obif*, *PLZF*, and *Sox9* primer sets following the previous reports [6, 21].

### Immunohistochemistry

Mouse testes were perfusion-fixed in 4% paraformaldehyde in PBS, embedded in TissueTec OCT compound, frozen, and sectioned. Frozen 10 μm sections on slides were dried for 30 min at room temperature, rehydrated in PBS for 5 min, incubated with blocking solution (10% newborn calf serum/0.5% Triton X-100 in PBS) for 1 h, and then incubated with the primary antibodies for 4 h at room temperature. Slides were washed three times with PBS for 10 min and incubated with a secondary antibody for 2 h at room temperature. The specimens were observed under a laser confocal microscope (LSM700, CarlZeiss). Rabbit anti-mouse SPACA1 polyclonal antibody was a gift from Dr. M. Ikawa (1:1000, Research Institute for Microbial Diseases, Osaka University, Osaka, Japan). Cy3-conjugated IgG (1:500, Jackson ImmunoResearch Laboratories) was used as a secondary antibody.

### Fertility test

Male WT and *Obif* ^−/−^ mice at 12 wks were obtained from same colony. They were housed with ICR strain female WT mice at 10 wks, one breeding pair per cage, for 4 weeks, and the numbers of litters and pups, as well as the time to first litter were documented. Male mice were euthanized at the end of the breeding period.

### Murine sperm counts and hormone measurement

Caudal epididymides were minced in 1 ml PBS and the number of cells released was counted after 1 h. The total sperm count was assessed in final suspension by using a hemocytometer [44]. The level of testosterone in serum samples was measured by ASKA Pharmaceutical Medical Co. Ltd. (Kawasaki, Japan) using LC-MS/MS (Liquid Chromatography—Tandem Mass Spectrometry).

### Serum biological measurements

The levels of phosphate and calcium in serum samples were measured by CLEA Japan (Tokyo, Japan). Serum 25-OH Vitamin D_3_ was measured by a Vitamin D EIA kit (Cayman Chemical, Ann Arbor, Michigan, USA). Serum osteocalcin was measured by a Mouse Osteocalcin EIA Kit (Biomedical Technologies, Stoughton, MA, USA).

### Statistics

Statistical analysis was performed using Student’s t test for comparisons between two groups, unless otherwise described. All data are expressed as mean ± SEM.

## Supporting Information

S1 Fig O-glycosylation of mObif protein had no effect on mineralization of MC3T3-E1 cells. Constructs of FLAG-tagged Nolan-GFP retrovirus vector (GFP), or FLAG-tagged mObif with or without mutation(s) (wild-type (WT), S36A, or S36A/S43A/T54A/ T60A/T67A (ALL)) were transfected into Phoenix cells. The Phoenix cells were cultured for 48 h. MC3T3-E1 cells were infected with supernatant containing the virus and cultured under mineralizing conditions. After 21 days, cells were stained with Alizarin red. Alizarin red staining was eluted with 28% Ammonia Solution and the acid soluble solution measured at OD 415nm. GFP, FLAG-tagged Nolan-GFP; mObif-WT, FLAG-tagged mObif Nolan-GFP; S36A, FLAG-tagged mObif-S36A Nolan-GFP; ALL, FLAG-tagged mObif-S36A/S43A/T54A/T60A/T67A Nolan-GFP. Error bars show the SEM (n = 4). *P < 0.05.
(TIF)

S2 Fig Serum biological measurements in *Obif* ^−/−^ mice. Calcuim **(A)**, Phosphate **(B)**, 25-OH Vitamin D_3_ **(C)**, and osteocalcin **(D)**. Ca, Calcium; IP, Inorganic phosphorus. Error bars show the SEM (n = 6). *P < 0.05.
(TIF)

S3 Fig Phenotype analysis in various tissues in *Obif* ^−/−^ mice. **(A)** RT-PCR analysis of *Obif* mRNA in various tissues isolated from wild-type mice at 4 wks. Intron-spanning primer sets of *Obif* or *β*-actin for RT-PCR generated 290 bp or 202 bp bands, respectively. **(B-G)** Histological analysis of *Obif* ^−/−^ mouse tissues. Toluidine blue staining of brain (hippocampus and cerebellum) sections from wild-type and *Obif* ^−/−^ mice at 12 wks (**B**). Hematoxylin and eosin (H&E) staining of heart, lung, spleen, skeletal muscle, and ovary sections from wild-type and *Obif* ^−/−^ mice at 8 wks (**C-G**). Histological features of the tissues from *Obif* ^−/−^ mice were unaltered compared with those from wild-type mice. Scale bars represent 500 μm **(B, C, E, F, and G)** and 100 μm **(D)**.
(TIF)

S4 Fig Obif expression in the developing mouse testis at P14. **(A-G)** *In situ* hybridization analysis for testicular sections. *Obif* antisense probe **(A)** and *Obif* sense probe **(B)** were used. Following testicular differentiation and cellular marker probes were used; *PLZF*, *Promyelocytic leukemia zinc-finger*, a marker for spermatogonia **(C)**, *Acrosin*, a marker for pachytene spermatocytes and round spermatids **(D)**, *Calmegin*, a marker for pachytene spermatocytes and spermatids **(E)**, *3β-HSD*, *3β-hydroxysteroid dehydrogenase/Δ5–Δ4 isomerase*, a marker for Leydig cells **(F)**, and *Sox9*, *SRY (sex determining region Y)-box 9*, a marker for Sertoli cells **(G)**. Scale bar represents 100 μm.
(TIF)
